# Antioxidant Actions of Thymoquinone, Silymarin, and Curcumin on Experimental Aortic Ischemia-Reperfusion Model in Wistar Albino Rats

**DOI:** 10.21470/1678-9741-2021-0462

**Published:** 2022

**Authors:** Mustafa Yardımcı, Mustafa Göz, Mehmet Salih Aydın, Nazım Kankılıç, Ebru Temiz

**Affiliations:** 1 Department of Cardiovascular Surgery, Medical Faculty, Harran University, Şanliurfa, Turkey.; 2 Department of Biochemistry, Medical Promotion and Marketing Program, Health Services Vocational School, Harran University, Şanliurfa, Turkey.

**Keywords:** Oxidative Stress, Reference Parameters, Reperfusion, Heart Ventricules, Ischemia, Aorta

## Abstract

**Introduction:**

Medical improvements are needed to prevent ischemia-reperfusion injury in thoracoabdominal aortic surgery. The aim of this study was to determine the antioxidant effects of thymoquinone, silymarin, and curcumin against ischemia-reperfusion injury associated with abdominal aorta.

**Methods:**

Twenty-five Wistar albino rats were included in the study. Sham, control, and treatment (thymoquinone, silymarin, and curcumin) groups were set in equal numbers. Ischemia-reperfusion was applied by clamping (120 minutes) and de-clamping (60 minutes) the infrarenal aorta of all groups, except the sham group. Before reperfusion, thymoquinone, silymarin, and curcumin were given intraperitoneally to the treatment groups. After reperfusion, blood samples were taken from the right ventricle. Total antioxidant status (TAS), total oxidant status (TOS), and oxidative stress index (OSI) were studied in serum samples and histopathological examination was performed on the gastrocnemius muscle.

**Results:**

There was a significant difference in TOS and OSI values between the control and sham groups. Both values were found higher in the control group than in the sham group (P<0.05). OSI values were found to be lower in the thymoquinone group compared to the control group (P<0.05). All three parameters were found to be lower in the silymarin group than in the control group (P<0.05). TAS and TOS levels were found to be higher in the curcumin group than in the control group (P<0.05). There was no histopathological difference between the groups.

**Conclusion:**

Silymarin and thymoquinone administration decreases oxidative stress in experimental aortic ischemia-reperfusion injury. Antioxidant effect of curcumin was lower than silymarin and thymoquinone.

**Table:** 

Abbreviations, Acronyms & Symbols			
AAA	= Abdominal aortic aneurysms	IAA	= Infrarenal abdominal aorta
AU	= Arbitrary units	NF-κB	= Nuclear factor kappa B
CAT	= Catalase	NO	= Nitric oxide
COX-2	= Cyclooxygenase-2	OSI	= Oxidative stress index
DMSO	= Dimethyl sulfoxide	PS	= Physiological serum
GPx	= Glutathione peroxidase	ROS	= Reactive oxidant species
GR	= Glutathione reductase	SOD	= Superoxide dismutase
GSH	= Glutathione (or γ-glutamylcysteinylglycine)	TAS	= Total antioxidant status
GST	= Glutathione S-transferase	TOS	= Total oxidant status
I-R	= Ischemia-reperfusion		

## INTRODUCTION

There is a complex structure including ischemia-reperfusion (I-R) injury, intracellular damage, and harmful inflammatory response damage. Anoxic cell damage is dominant in the ischemic phase^[1]^. Ischemia causes depletion of tissue energy sources, activation of proteases, and calcium flow into the ischemic cell^[2]^. Decreased mitochondrial adenosine triphosphate production disrupts cellular ionic balance with loss of selective permeability of the cell membrane and activation of hydrolases. The inflammatory response begins with reperfusion. This disrupts the microcirculation and causes apoptosis and necrosis^[2]^. Reactive oxidant species (ROS), complement activation, leukocyte-endothelial-platelet adhesion/interaction, increased microvascular permeability, endothelial-dependent vasodilatation dysfunction, and increased inflammatory molecule (cytokine, chemokine) constitute the main mechanisms in I-R injury^[3-5]^.

Aerobic creatures have several mechanisms to counter ROS damage. The basic system for protection from ROS damage is the enzymatic system that prevents oxidation. The other system is non-enzymatic antioxidant compounds^[6]^. The enzymatic system tries to eliminate all radicals, but the second line of the defense system activates nonenzymatic antioxidant compounds if the oxidative stress is higher than the capacity of the defense mechanism. Antioxidant enzymes are catalase (CAT), superoxide dismutase (SOD), glutathione reductase (GR), glutathione peroxidase (GPx), and glutathione S-transferase (GST). Non-enzymatic antioxidants include reduced glutathione (or γ-glutamylcysteinylglycine) (GSH), vitamin E, vitamin C, thioredoxin, etc.^[7]^. Antioxidant defense components containing thiol groups constitute the secondary defense line in protecting against ROS-mediated oxidative damage^[8]^. GSH is a non-protein thiol compound involved in antioxidant defense^[9]^. Synthesis enzymes such as γ-glutamylcysteinesynthetase, glutathione synthetase, enzymes involved in antioxidation such as GPx, GR, and GST, and enzymes involved in intra/intercellular transport such as γ-glutamyltranspeptidase all constitute the glutathione redox system^[10]^.

Interventions in the repair of abdominal aortic aneurysms (AAA) are associated with severe morbidity and mortality despite modern approaches to organ preservation^[11,12]^. Systemic inflammatory response syndrome and multiple organ failure due to ischemia injury lead to local or distant tissue damage and increase the risk of mortality in ruptured AAA^[13]^. Applications and treatments to be developed in these pathologies may reduce mortality and morbidity rates by reducing I-R damage.

Antioxidant therapies have been used in many studies to prevent or reduce the effect of I-R damage. These treatments are generally known for its effects on the anti-inflammatory response, calcium channels, and SOD activities^[14]^.

### Thymoquinone

Thymoquinone is a monoterpene quinone compound obtained from the *Nigella sativa* plant^[15-17]^. It is accompanied by thymol and ditimoquinone compounds even though the main component of *N. sativa* is thymoquinone^[17]^. There are *in vitro* and *in vivo* studies on the antioxidant, antihepatotoxic, anti-inflammatory and analgesic, anticarcinogenic, and antimicrobial properties of thymoquinone^[15-18]^. Thymoquinone, a powerful OH radical scavenger, shows its antioxidant properties by increasing antioxidant enzyme activities such as SOD, CAT, GPx, and GSH amount^[17]^.

### Silymarin

*Silybum marianum* (L.) Gaertner (milk thistle) is a plant of the Asteraceae family. Silymarin is a complex compound derived from the seeds of the plant *S. marianum*. The main component is silybin. Silymarin is a flavonolignan compound^[19]^. It consists of isosilybin, silychristin, silydianin, and taxifolin, which is a flavonoid structure, among other flavonolignans. Silybin is known to be the most effective antihepatotoxic agent in the silymarin complex^[19]^. It has its antihepatotoxic effect with free radical scavenger, ROS, and lipid peroxidation reduction, nuclear factor kappa B (NF-κB) and nitric oxide (NO) modulation, and a decrease in cyclooxygenase-2 (COX-2) expression^[19,20]^.

### Curcumin

Curcumin is a bright yellow polyphenol-derived herbal extract obtained from the root part of the plant *Curcuma longa*, known as turmeric saffron. Curcumin has many pharmacological properties, including anti-inflammatory, antioxidant, anticancer, antiatherosclerotic, and antimicrobial properties. It was determined that many molecules such as transcription factors, growth factors, enzymes, cytokines, and protein kinases were targeted^[21,22]^.

In this study, the effects of thymoquinone, silymarin, and curcumin on oxidative stress parameters (total antioxidant status [TAS], total oxidant status [TOS], oxidative stress index [OSI]) were investigated in experimental abdominal aorta I-R injury.

## METHODS

Our study was carried out in accordance with the Regulation on the Working Procedures and Principles of Animal Experiments Ethics Committees published by the Ministry of Environment and Forestry in the Official Gazette dated 6 July 2006 and numbered 2622 and the Harran University Animal Experiments Local Ethics Committee Directive after the approval of Dollvet Ethics Committee (ethics committee approval dated 07/12/2014 and numbered 2014/62).

### Study Groups and Protocol

Twenty-five Wistar albino rats with an average weight of 250-300 g were randomly divided into five equal groups. The rats were kept at room temperature, 12 hours of light, and 12 hours of darkness before the study. All rats were fed tap water and standard rat feed under standard conditions. Feeding of all rats was discontinued eight hours before the intervention. The groups were:

**Group 1** (sham, n=5): No procedures other than anesthesia were performed during the study. Tissue and blood samples were collected appropriately at the time corresponding to the end of the I-R period.

**Group 2** (control, I-R, n=5): 120-minute ischemia and 60-minute reperfusion were applied to the infrarenal abdominal aorta (IAA), and no medication was given after anesthesia. Tissue and blood samples were collected appropriately at the time corresponding to the end of the I-R period.

**Group 3** (I-R + thymoquinone, n=5): 120-minute ischemia was applied to the IAA after anesthesia. Thymoquinone (20 mg/kg)^[23]^ was administered intraperitoneally and 60-minute reperfusion was applied immediately after ischemia was terminated. Tissue and blood samples were collected appropriately at the time corresponding to the end of the I-R period.

**Group 4** (I-R + silymarin, n=5): 120-minute ischemia was applied to the IAA after anesthesia. Silymarin (200 mg/kg)^[24]^ was administered intraperitoneally and 60-minute reperfusion was applied immediately after ischemia was terminated. Tissue and blood samples were collected appropriately at the time corresponding to the end of the I-R period.

**Group 5** (I-R + curcumin, n=5): 120-minute ischemia was applied to the IAA after anesthesia. Curcumin (200 mg/kg)^[25]^ was administered intraperitoneally and 60-minute reperfusion was applied immediately after ischemia was terminated. Tissue and blood samples were collected appropriately at the time corresponding to the end of the I-R period.

### Ischemia-Reperfusion Damage Model

Ketamine (87 mg/kg, intraperitoneally) (Ketalar; Parke Davis, Eczacıbaşı, Istanbul, Turkey) and xylazine (13 mg/kg) (Rompun; Bayer AG, Leverkusen, Germany) were administered to all rats used in the experiment after eight hours of fasting. If necessary, an additional dose was planned, once during the experiment. A midline laparotomy was performed on rats whose skin was aseptically prepared. IAA was carefully explored after the intestines were removed with the help of wet gauze. A non-traumatic microvascular clamp was placed in the IAA. The microvascular clamp in the IAA was removed 120 minutes later, and reperfusion was achieved for 60 minutes. Aortic ischemia was confirmed by the loss of pulsation in the distal aorta during the clamping procedure, and aortic reperfusion was confirmed by the return of pulsation in the distal aorta after the clamp was removed. Laparotomy and abdominal aortic procedures were performed in equal time (120 minutes) in rats from the control group. Physiological serum (PS) was applied to the peritoneal cavity in the periods after clamping and removal of the IAA, and the abdominal incision was temporarily closed by wrapping with a wet gauze cloth in order to minimize heat and fluid loss from the peritoneal cavity in the I-R periods. The median laparotomy incision was advanced upwards and opened from the mediastinum, the heart was reached, and blood was taken from the right ventricular cavity with the help of a 5-cc injector in all rats at the end of the reperfusion period. Afterward, the right gastrocnemius muscle tissue sample was taken. Muscle tissue samples were stored in 10% formaldehyde solution until immunohistochemical and hematoxylin-eosin evaluation were performed. Blood from rats was centrifuged at 4000 RPM for 10 minutes and rat plasma samples were stored at-20°C until biochemical analyses were performed.

### Preparation of Thymoquinone, Silymarin, and Curcumin

Thymoquinone and silymarin were prepared using PS, and curcumin was prepared with 1% dimethyl sulfoxide (DMSO) (Sigma Chemical Company, Germany). Prepared treatments were administered by intraperitoneal injection.

### Histopathologic Examination of Gastrocnemius Muscle

Muscle tissues were fixed separately in 10% buffered neutral formaldehyde solution (Sigma Chemical Company, Germany) for histopathological examination. Samples were embedded in paraffin blocks, and 5-micrometer sections were taken. They were stained with hematoxylin-eosin stain (Sigma Chemical Company, Germany). 20 lens magnification was used (Olympus BX51 TF, United States of America). Interstitial edema, muscle fiber degeneration, nuclear centralization, inflammatory cell infiltration, disorganization, and necrosis were determined as histopathological parameters. The scoring (none: 0, yes: 1, significant: 2) was arranged for each histopathological parameter^[26]^. Damage was estimated by summing up the parameters. The histopathological score was determined and recorded for each sample.

### Total Antioxidant Status Measurement

TAS level of the samples was measured using Rel Assay commercial kits (Rel Assay Diagnostics, Gaziantep, Turkey). The measurement method was based on the fact that all antioxidant molecules in the sample reduced the colored 2,2'-azino-bis (3-ethylbenzothiazoline-6-sulfonic acid) (or ABTS) cationic radical, and the colored radical was decolorized in proportion to the total concentrations of antioxidant molecules. Trolox, a water-soluble analog of vitamin E, was used as the calibrator. The results were expressed as mmol Trolox Equivalent/L (mmol Trolox equivalent/gr protein)^[27]^.

### Total Oxidant Status Measurement

TOS level of the samples was measured using Rel Assay commercial kits (Rel Assay Diagnostics, Gaziantep, Turkey). Measurement was performed by a colorimetric method based on the cumulative oxidation of the oxidant molecules contained in the samples to the ferric ion as expressed in the working principle of the test. The results are expressed as µmol H2O2 Equivalent/L (µmol H2O2 equivalent/gr protein)^[28]^.

### Oxidative Stress Index Calculation

TAS levels were converted to µmol Trolox equivalent/gr protein in the OSI calculation. The ratio of TOS levels contained in the samples to TAS levels was determined as OSI^[25]^. The results were expressed as arbitrary units. OSI = (TOS, µmol H_2_O_2_ equivalent/gr protein)/(TAS, µmol Trolox equivalent/gr protein) × 100.

### Statistical Analysis

Statistical results of the biochemical analysis were calculated in the SPSS Inc. Released 2008, SPSS Statistics for Windows, version 17.0, Chicago: SPSS Inc. package software. The normal distribution was determined by Kolmogorov-Smirnov test. Kruskal-Wallis test was used to examine whether there was a difference between more than two independent groups in terms of a continuous variable. Mann-Whitney U test was used to test whether there was a difference between two groups independent of a continuous variable in the parameters that were important in the Kruskal-Wallis test. The confidence interval was accepted as 95% throughout the analyses. *P*<0.05 was considered statistically significant.

## RESULTS

### Effect of Thymoquinone, Silymarin, and Curcumin on TAS, TOS, and OSI values

**Fig. 1:**
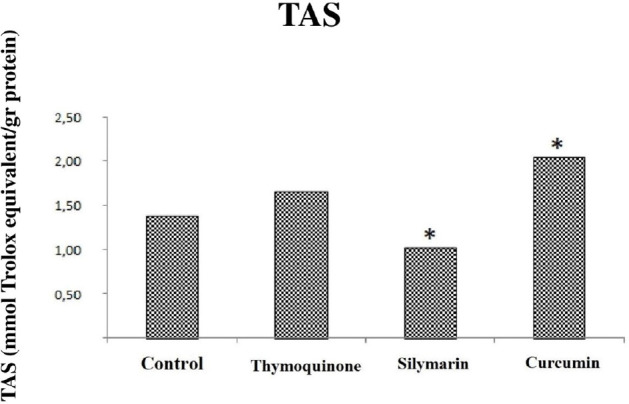
Comparison of total antioxidant status (TAS) values between groups. Control = ischemia-reperfusion (I-R) group; Thymoquinone = thymoquinone + I-R group; Silymarin = silymarin + I-R group; Curcumin = curcumin + I-R group. *=statistically significant

**Fig. 2:**
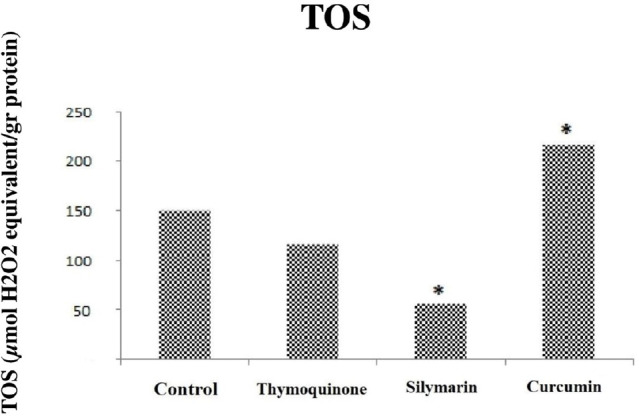
Comparison of total oxidant status (TOS) values between groups. Control = ischemia-reperfusion (I-R) group; Thymoquinone = thymoquinone + I-R group; Silymarin = silymarin + I-R group; Curcumin = curcumin + I-R group. *=statistically significant

**Fig. 3:**
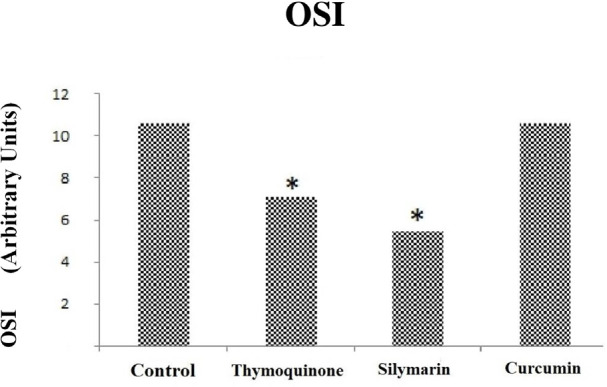
Comparison of oxidative stress index (OSI) values between groups. Control = ischemia-reperfusion (I-R) group; Thymoquinone = thymoquinone + I-R group; Silymarin = silymarin + I-R group; Curcumin = curcumin + I-R group. *=statistically significant

The effects of thymoquinone, silymarin, and curcumin on TAS, TOS, and OSI parameters are shown in Table 1. There was no difference in TAS values in the sham and control groups, but there was a significant difference in TOS and OSI values (*P*<0.05). The oxidant capacity was higher in the control group than in the sham group (Table 1). OSI values were found to be low in the thymoquinone group (*P*=0.009) (Figure 3), while there was no difference between the thymoquinone group and the control group in terms of TAS and TOS values (*P*>0.05) (*P*=0.175, *P*=0.347) (Figures 1 and 2). TAS, TOS, and OSI were found to be low in the silymarin-treated group when this group and the control group were compared (*P*=0.009) (Figures 1, 2, and 3). The levels of TAS and TOS were higher in the curcumin group than in the control group (*P*<0.05) (Figures 1 and 2) and OSI values were similar (Figure 3), unlike thymoquinone and silymarin groups. Regarding TAS values, there was no change in the thymoquinone group compared to the control group, whereas there was a 27% decrease in the silymarin group and a 48% increase in the curcumin group. Regarding TOS values, there was no change in the thymoquinone group compared to the control group, whereas there was a 63% decrease in the silymarin group and a 45% increase in the curcumin group. And regarding OSI values, there was a 33% decrease in the thymoquinone group, 49% decrease in the silymarin group, and no change in the curcumin group compared to the control group.

**Table 1:** Total antioxidant status (TAS), total oxidant status (TOS), and oxidative stress index (OSI) measurements.

	TAS (Trolox equivalent/gr protein)	TOS (µmol H ^2^O^^2^^ equivalent/gr protein)	OSI (AU)
Sham (N=5)	1.47±0.14	77.11±10.08	5.22±0.36
Control (N=5)	1.39±0.08	150.16±19.96^#^	10.63±0.90^#^
Thymoquinone (N=5)	1.66±0.16	117.12±11.09	7.09±0.25*
Silymarin (N=5)	1.02±0.07**	55.81±7.72**	5.45±0.68**
Curcumin (N=5)	2.05±0.09***	217.23±32.01***	10.62±1.46

AU=arbitrary units

# *P*<0.05; control *vs.* sham (Mann-Whitney U test)

* *P*<0.05; control *vs.* thymoquinone (Mann-Whitney U test)

** *P*<0.05; control *vs.* silymarin (Mann-Whitney U test)

*** *P*<0.05; control *vs.* curcumin (Mann-Whitney U test)

### Histopathologic Evaluation of Gastrocnemius Muscle

No statistical difference was observed between the groups according to the damage scores determined by the data obtained from histopathological images.

## DISCUSSION

One of the most important problems in the I-R mechanism is the total reactive oxygen products and the total antioxidant capacity against it^[3-5]^. There are many methods to measure oxidant and antioxidant capacity. Among these, TAS and TOS are easy, reliable, sensitive, and inexpensive methods that can be performed fully automatically for the measurement of total oxidant and antioxidant capacities^[27,28]^. Therefore, all three parameters showing oxidant and antioxidant capacities were quickly, easily, and reliably studied in our study. As a result of these evaluations, the oxidant capacity was found to be higher in the ischemia-treated control group compared to the sham group, which was to be expected. A significant difference was observed on the OSI value in the thymoquinone group compared to the control group (even though a decrease in TOS values was observed, no significant statistical difference was observed). This result indicates that thymoquinone activates antioxidant systems in I-R injury. This effect is similar to the presented in the study of Gökçe et al.^[29]^, showing that thymoquinone reduces TOS and OSI values in I-R injury in rat testicular tissue. Thymoquinone probably performs its antioxidant activity by increasing antioxidant enzyme activities (SOD, CAT, GPx) and GSH levels^[30]^. Thymoquinone can also reduce the inflammatory activity that occurs with I-R. This demonstrates its efficacy by reducing inflammatory cytokines and reducing tumor necrosis factor-α^[31]^. This anti-inflammatory activity caused by thymoquinone causes a decrease in the amount of ROS formed.

Silymarin was found to be the herbal treatment that showed the best antioxidant activity in our study. Silymarin, which significantly reduced all the TAS, TOS, and OSI values, was thought to have better antioxidant properties than thymoquinone and curcumin. Silymarin has been used mainly in the treatment of liver and gastrointestinal diseases and is still used today against cirrhosis, chronic hepatitis, alcohol-related liver diseases, and various environmental toxic substances^[24,32,33]^. Two main mechanisms are proposed to explain the hepatoprotective property of silymarin. The first is based on its antioxidant effect due to its strong free radical scavenger, ROS, and lipid peroxidation reducing properties. The second is its anti-inflammatory and antiapoptotic mechanisms due to NF-κB modulation, NO modulation, and a decrease in COX-2 expression^[20,32]^. GSH levels have also been reported to increase similarly to thymoquinone^[34]^. Curcumin was found to reduce oxidative stress in rat ovary^[35]^, prevent histopathological damage in mesenteric I-R injury and intestinal tissue, reduce lung damage, and reduce malondialdehyde, and other oxidative stress parameters^[25,36]^. It reduces endothelial dysfunction^[37]^, myocardial I-R injury^[38]^, and acts as a cardioprotective antioxidant^[39,40]^. Curcumin exhibits anti-inflammatory activity similarly to thymoquinone^[22]^ and inhibits NF-κB metabolism similarly to silymarin^[41]^. Its efficacy in our study lagged behind the other two treatments even though curcumin was recognized as an effective antioxidant. We think that DMSO, which is used as a solvent in this effect of curcumin, plays a role as both prooxidant and antioxidant^[42,43]^.

Distal organ damage was tried to be determined by gastrocnemius muscle histopathology. There were no histopathological changes in gastrocnemius muscle tissue for all groups in our study. We think that this is due to ischemia and reperfusion times and the insensitivity of gastrocnemius muscle tissue to I-R damage (muscle tissue is more resistant to I-R damage compared to other tissues). It was reported that cell death would not be seen by light microscopy until 10-12 hours after complete ischemia^[44]^. This supports our idea that the absence of histopathological change is due to low I-R times.

There are many studies related to herbal treatment methods and antioxidant studies when the literature is examined. However, the number of studies comparing these treatments is fairly limited. Therefore, our article will guide the extent to which the antioxidant activity of I-R after clamping of the abdominal aorta changes with thymoquinone, silymarin, and curcumin.

### Limitations

This study has several limitations. The first relates to the administration of medications. Thymoquinone, silymarin, and curcumin were administered intraperitoneally. It is not foreseen how the effects of administering it in different ways (intravenous, oral) will be and how it will affect the effectiveness. The second is the effect of medication solvents in the study on oxidant and antioxidant processes. Third is that basic biochemical parameters and especially inflammatory parameters are not studied due to the anti-inflammatory efficacy of all three treatments. And the fourth and last limitation is that histopathological samples are not taken from different tissues, and changes in other tissues are not seen.

## CONCLUSION

Thymoquinone and silymarin significantly reduce oxidative stress in the I-R injury model applied to the abdominal aorta. Silymarin’s antioxidant activity is much more effective compared to the other two agents. Curcumin’s antioxidant effect is much lower compared to the other two agents. Histopathological changes in peripheral end organ damage are thought to occur after longer I-R periods. Further studies are needed in the future to better understand the effects of thymoquinone, silymarin, and curcumin.
